# Unraveling the Immune Regulatory Functions of USP5: Implications for Disease Therapy

**DOI:** 10.3390/biom14060683

**Published:** 2024-06-12

**Authors:** Jinyi Gu, Changshun Chen, Pu He, Yunjie Du, Bingdong Zhu

**Affiliations:** 1 Institute of Pathogen Biology, School of Basic Medical Sciences, Lanzhou University, Lanzhou 730030, China; janegu@ynu.edu.cn (J.G.); hep19@lzu.edu.cn (P.H.); 120220905730@lzu.edu.cn (Y.D.); 2 Gansu Provincial Key Laboratory of Evidence Based Medicine and Clinical Translation, Lanzhou 730030, China; 3 Clinical Laboratory, Affiliated Hospital of Yunnan University, Kunming 650032, China; 4 Department of Orthopedics, Lanzhou University Second Hospital, Lanzhou 730030, China; chencs666@ynu.edu.cn; 5 Department of Orthopedics and Trauma Surgery, Affiliated Hospital of Yunnan University, Kunming 650032, China

**Keywords:** USP5, deubiquitinating enzyme, immune regulation, targeted therapy

## Abstract

Ubiquitin-specific protease 5 (USP5) belongs to the ubiquitin-specific protease (USP) family, which uniquely recognizes unanchored polyubiquitin chains to maintain the homeostasis of monoubiquitin chains. USP5 participates in a wide range of cellular processes by specifically cleaving isopeptide bonds between ubiquitin and substrate proteins or ubiquitin itself. In the process of immune regulation, USP5 affects important cellular signaling pathways, such as NF-κB, Wnt/β-catenin, and IFN, by regulating ubiquitin-dependent protein degradation. These pathways play important roles in immune regulation and inflammatory responses. In addition, USP5 regulates the activity and function of immunomodulatory signaling pathways via the deubiquitination of key proteins, thereby affecting the activity of immune cells and the regulation of immune responses. In the present review, the structure and function of USP5, its role in immune regulation, and the mechanism by which USP5 affects the development of diseases by regulating immune signaling pathways are comprehensively overviewed. In addition, we also introduce the latest research progress of targeting USP5 in the treatment of related diseases, calling for an interdisciplinary approach to explore the therapeutic potential of targeting USP5 in immune regulation.

## 1. Introduction

Ubiquitin-specific protease 5 (USP5), also known as isopeptidase T (ISOT), is a deubiquitinating enzyme belonging to the ubiquitin-specific protease (USP) family originally identified by Wilkinson et al. [1,2]. This protein is encoded by the human USP5 gene located on chromosome 12p13 and is predominantly localized in the cytoplasm and nucleoplasm [3,4]. It uniquely recognizes unanchored (not bound to the target protein) polyubiquitin chains to maintain the homeostasis of monoubiquitin chains [2,5]. A growing body of evidence suggests that USP5 plays a key role in cells. By specifically cleaving the isopeptide bonds between ubiquitin and substrate proteins or ubiquitin itself, it participates in a wide range of cellular processes, including protein degradation, signal transduction, cell cycle control, DNA repair, stress responses, and inflammation [2,6,7,8,9,10]. In addition to its role in basic cell biology, the abnormal expression and dysfunction of USP5 are also associated with the occurrence and development of a variety of diseases, including tumors, pathological pain, developmental abnormalities, inflammatory diseases, and viral infections, highlighting the significance of this protein as a potential therapeutic target [2,10].

Immune regulation plays a crucial role in maintaining homeostasis, preventing infectious diseases, and inhibiting tumor development [11,12,13]. Immunomodulation-related signaling pathways can regulate the activation, proliferation, differentiation, and function of immune cells, thereby affecting the strength and type of immune response, leading to the occurrence and progression of diseases [14,15,16,17]. For example, the immunomodulatory coinhibitory molecules CTLA4 and PD-1 play a major role in regulating T-cell receptor signaling and are critical for maintaining peripheral self-tolerance and modulating immune responses [17]. These inhibitory signals have active roles in effectors and regulatory immune cells at multiple sites, and their important role in human immune homeostasis has been demonstrated by the autoimmune-related side effects in cancer clinical trials with PD-1 or CTLA4-blocking antibodies [14]. Yan et al. showed that JAK/STAT signaling plays a key role in the action of a variety of cytokines and interferon (IFN) and is essential for the development and function of innate and adaptive immunity [15]. The role of Notch signaling in the immune system, especially in acute and chronic inflammation, reveals the critical regulatory role of the Notch pathway in innate immunity and inflammatory responses and the potential impact of this regulation on the pathogenesis and treatment of inflammatory diseases [16]. In addition, Hammond et al. showed that in Alzheimer’s disease, studies of immune signaling pathways, such as TGF-β, the complement system, and TREM2, have revealed novel functions for them in healthy and disease conditions [17].

Although direct studies specifically linking USP5 with immune system regulation are sparse, the roles of ubiquitination and deubiquitination in immune regulation are well documented, indirectly suggesting how USP5 could be involved [18,19,20]. In the process of immune regulation, USP5 affects a variety of cellular signaling pathways by regulating ubiquitin-dependent protein degradation, such as NF-κB and Wnt/β-catenin signaling pathways, which play important roles in immune regulation and inflammatory responses [20,21]. In addition, USP5 may affect the activity and function of key proteins in the immune regulatory signaling pathway by regulating protein deubiquitination and then affect the activity of immune cells and the regulation of immune responses [8,19,22,23]. This potential association may provide a new perspective for studying the mechanism of immune regulation and contribute to a deeper understanding of the regulatory mechanism of immune regulatory and signaling pathways.

In this article, the structure and function of USP5, the role of USP5 in immune regulation, and the mechanism of USP5 affecting the development of diseases by regulating immune signaling pathways were comprehensively reviewed. In particular, it provides valuable insights into the complex mechanisms by which USP5 participates in a wide range of cellular processes through the regulation of immune signaling pathways to influence disease development, underscoring the importance of this protein as a potential therapeutic target. In addition, we also introduced the latest research progress of targeting USP5 in the treatment of related diseases, calling for an interdisciplinary approach to explore the therapeutic potential of targeting USP5 in immune regulation.

## 2. Structure and Function of USP5

### 2.1. Overview of the USP Family

Tobias et al. first discovered and cloned USPs in Saccharomyces cerevisiae in 1991 [24]. The USP family, with more than 50 members, constitutes the largest subfamily of DUBs [25,26]. USPs share a high degree of homology in the catalytic domain, and the catalytic core is a catalytic triad consisting of three very conserved motifs: the catalytic Cys residue, catalytic His residue, and catalytic Asp/Asn residue [25,27,28]. This catalytic triad is located within a highly conserved USP pocket, similar to an open hand with “thumb” (Cys), “palm” (His/Asp), and “finger” subdomains, with the catalytic site located between the palm and thumb domains, and the finger domain being responsible for interacting with distal ubiquitin [27,28,29,30]. However, studies have found that USPs such as USP16, USP30, USP39, USP45, and USP52 lack catalytic residues [26,28]. In addition, due to the large regulatory sequences scattering between the conserved motifs, the size of the USP catalytic domain can vary between 295 and 850 amino acids. Inside the catalytic domain structure of 27 USPs, there are 300–400 amino acids, while in the catalytic domain structure of 29 USPs, there are 400–850 amino acids [27,31]. In addition to the catalytic domain, USPs also possess domains for subcellular localization, substrate specificity, zinc binding, and ubiquitin recognition [25,27,28,31]. For example, USP3, USP5, USP39, USP44, USP45, USP49, and USP51 have zinc-finger USP domains; USP25 and USP37 show the ubiquitin interaction motif; USP4, USP11, USP15, USP20, USP33, and USP48 have USPs domains (DUSP); and USP4, USP7, USP14, USP32, USP47, and USP48 display ubiquitin-like domains that can be located either within or outside the catalytic domain [26,32,33,34]. However, despite their relatively diverse structures, most USPs share a common feature that the modular character of the USP enzyme imparts binding selectivity to substrates and ubiquitin chains, as well as a typical conformational change upon ubiquitin binding that drives a transition from an inactive form to a catalytically active state [27,29,32,35]. The proper binding and catalysis of ubiquitin require an intact catalytic triad that rearranges to position the catalytic cysteine residue in range with the histidine residue [27,32,35]. As cysteine proteases, the catalytic capacity of USPs is mainly dependent on nucleophilic attack by the catalytic site of cysteine [29]. 

### 2.2. Structure and Catalytic Activity of USP5

**Figure 1:**
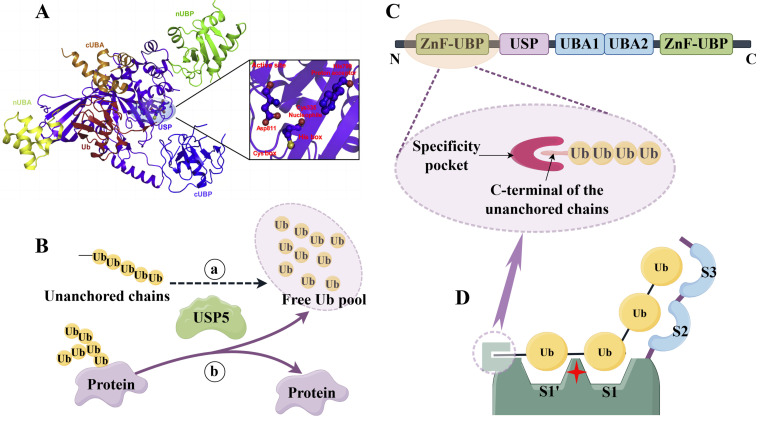
Structure and function of USP5. (**A**): 3D structure schematic of USP5 (PDB ID: 3ihp) [2]. (**B**): deubiquitination function of USP5. a: USP5 specifically recognizes unanchored ubiquitin chains to maintain the homeostasis of the free ubiquitin pool. b: USP5 specifically removes target protein ubiquitin modification to maintain protein homeostasis and signal transduction in cells. (**C**): ubiquitin-binding domains of USP5. (**D**): four ubiquitin-binding sites of USP5. Created using Figdraw.com (https://www.figdraw.com, accessed on 8 June 2004).

USP5, through its catalytic activity, plays a crucial role in the process of deubiquitination, which involves the cleavage of ubiquitin from ubiquitinated proteins. Structurally, USP5 consists of five separate domains (Figure 1A,C). Specifically, this includes a ubiquitin-specific protease domain containing the catalytic core (USP core domain), a C-terminal ZnF-UBP (cUBP), an N-terminal ZnF-UBP (nUBP), and two ubiquitin binding-related domains (UBA1 and UBA2) [2,10,19,36].

The USP core domain is responsible for the catalytic activity, which contains a catalytic core consisting of a Cys-box and His-box, which can specifically recognize and hydrolyze the isopeptide bond on the ubiquitin chain to initiate deubiquitination activity [2,37]. ZnF-UBP is one of the unique structural features of USP5, and its tight binding to the catalytic core is indispensable for USP5 to exert catalytic activity [10]. Additionally, nUBP contains a specific deep-binding pocket that recognizes ubiquitin activity and enhances proximal ubiquitin cleavage. This pocket is embedded in the C-terminal double glycine residue of the free polyubiquitin chain and is tightly bound to the catalytic core, which is essential for catalytic activity [38] (Figure 1C,D). However, Avvakumov et al., while exploring the structural features of USP5, identified two ZnF-UBP domains by determining the structure of full-length USP5 [38]. This finding suggests that USP5 uses multiple ZnF-UBP domains for substrate localization and core catalytic functions, whereas one of the known cUBP domains does not directly contribute to the active site, and its deletion does not significantly affect the rate of ubiquitin–AMC hydrolysis, suggesting that it may be primarily related to substrate localization and specificity [38,39]. Another study revealed the structure–activity relationship of USP5 and found that a chemical series can occupy the USP5 cUBP domain ubiquitin-binding site and thus competitively inhibit the catalytic activity of the enzyme, revealing the mechanism by which USP5 regulates its catalytic activity [40]. The exploration of the structure–activity relationship combined with the crystallographic characterization of ZnF-UBP binding to multiple ligands identified a compound that bound to USP5 ZnF-UBP with a KD value of 2.8 μM and inhibited the USP5 catalytic cleavage of dimeric ubiquitin substrates in vitro [40]. UBA1 and UBA2 bind to the third and fourth ubiquitin molecules in the linear and K48-linked polyubiquitin chains, respectively, and thus exert their regulatory functions [41,42]. In addition, the deubiquitination activity of USP5 can be activated by the active site modulation of its interacting proteins, such as USP1-related factor 1 (UAF1). This atypical cysteine protease modulates its active site conformation with UAF1 to achieve general base catalysis at a neutral pH, which plays an important role in its improved catalytic efficiency [43].

Most studies have reported on the removal of substrate ubiquitination by USP5 through its deubiquitination activity. Interestingly, USP5 can also enhance ubiquitin chains on its target proteins independently of its enzymatic activity [8,44]. For example, Liu et al. found that USP5 could link STUB1 to RIG-I and promote the formation of K48-linked ubiquitin chains on RIG-I [8]. The mechanism of action is that USP5 may help to increase the stability of E3 ligase or reduce the steric hindrance between the substrate and E3 ligase. In this process, the ubiquitin USP5 domain is very important for the enhancement of ubiquitin. Another study reported that USP5 could promote, but not cleave, the K48-linked ubiquitination level of NLRP3 and degrade NLRP3 [44]. The rationale is that USP5 may act as a key scaffold protein and recruit a specific E3 ligase to regulate NLRP3 inflammasome activation. However, the relationship between the structure of USP5 and this non-classical function that enhances the ubiquitin chain on its target protein, independent of its enzymatic activity, is currently unknown. 

### 2.3. Role of USP5 in Maintaining Protein Stability and Signaling

USP5 plays an important role in maintaining protein stability and signal transduction. In the regulation of protein stability, USP5 specifically recognizes unanchored polyubiquitin chains and cuts the isopeptide bond between the carboxyl end of proximal ubiquitin molecules and the lysine residue of distal ubiquitin molecules, resulting in the removal of ubiquitin from the ubiquitin chain to generate monoubiquitin molecules and maintain the homeostasis of the free ubiquitin pool [2,5] (Figure 1B). At the same time, USP5 also specifically removes the ubiquitination modification of the target protein and prevents its degradation, which plays an important role in maintaining the stability of the free ubiquitin pool [45] (Figure 1B). During Drosophila eye development, USP5 regulates protein stability by controlling apoptosis and Jun N-terminal kinase (JNK) pathway activation [46]. In addition, USP7, a deubiquitinase similar to USP5, was found to interact with the mixed lineage leukemia 5 protein (MLL5) to enhance its stability [47]. 

**Figure 2:**
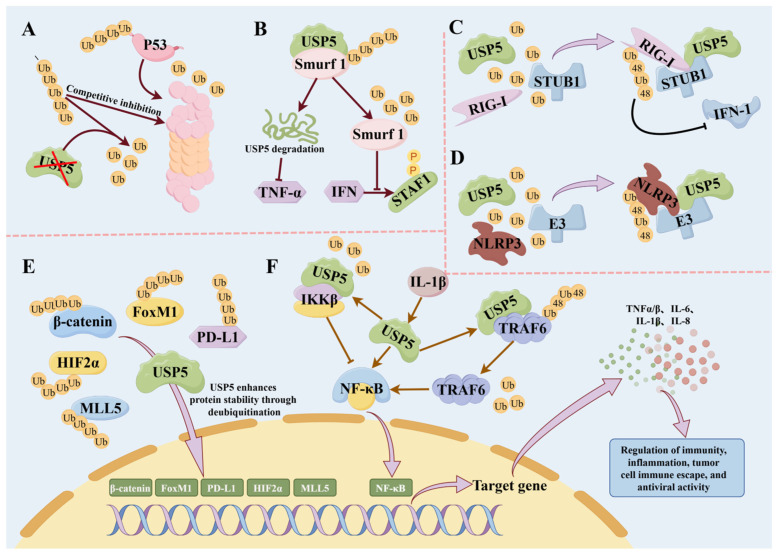
USP5 participates in immune regulation through a variety of signaling pathways. (**A**): knockdown of USP5, which allows the competitive binding of accumulated unanchored polyubiquitin and inhibits proteasomal recognition sites on P53, thereby inhibiting the proteasomal-mediated degradation of p53 and increasing its transcriptional activity to negatively regulate p53-related pathways. (**B**): Smurf1 interacts with USP5, promotes the degradation of USP5 through the ubiquitin protease pathway, and reduces the protein expression of USP5, thereby inhibiting the production of TNF-α. On the other hand, USP5 physically interacts with Smurf1 and inhibits the expression of IFN-activated antiviral genes by decreasing the level of polyubiquitination of Smurf1 and enhancing the stability and turnover capacity of Smurf1. (**C**): USP5 bridges STUB1 to RIG-I and promotes the formation of the K48-linked ubiquitin chains on RIG-I, thus facilitating the degradation of RIG-I and inhibiting type I IFN signaling. (**D**): USP5 selectively promotes K48-linked NLRP3 polyubiquitination by recruiting the E3 ligases MARCHF7 and mediates its degradation via the autophagy lysosome pathway. (**E**): USP5 enhances the stability of MLL5, HIF2α, β-catenin, FoxM1, PD-L1, and other proteins via deubiquitination. (**F**): On the one hand, USP5 interacts with IKKβ and prevents IKKβ ubiquitination, which in turn inhibits the NF-κB signaling pathway; on the other hand, USP5 promotes the NF-κB signaling pathway by deubiquitinating TRAF6. Created using Figdraw.com (https://www.figdraw.com, accessed on 8 June 2004).

In addition, the deubiquitination activity of USP5 has a regulatory effect on protein stability and degradation. USP5 is directly or indirectly involved in multiple signal transduction pathways via the deubiquitination of key signal proteins, thereby affecting intracellular signal transmission and function execution [2]. For example, the knockdown of USP5, which allows the competitive binding of accumulated unanchored polyubiquitin, inhibits proteasomal recognition sites on P53, thereby inhibiting the proteasomal-mediated degradation of p53 and increasing its transcriptional activity to negatively regulate p53-related pathways [48] (Figure 2A). USP5 can stabilize β-catenin via deubiquitination, leading to the nuclear accumulation and activation of β-catenin, and positively regulates the Wnt/β-catenin pathway [49,50] (Figure 2E). USP5 can negatively regulate the type I interferon signaling pathway by interacting with STUB1, the E3 ubiquitin ligase of RIG-I, to promote the ubiquitination of K48 of RIG-I and promote the degradation of RIG-I [8] (Figure 2C). In addition, USP5 negatively regulates the Notch and RTK signaling pathways by inhibiting Jun N-terminal kinase (JNK) [46,51,52].

These studies suggest that USP5, through its deubiquitinating enzyme activity, is involved in a variety of cellular functions and disease processes by regulating key nodes in protein stability and signaling. These findings highlight the importance of USP5 as a potential target for disease therapy.

## 3. Role of USP5 in Immune Regulation

### 3.1. USP5 Regulates Immune Cell Activation, Differentiation, and Function

Although the role of USP5 in the activation, differentiation, and function of immune cells has not been well studied, there is a particular lack of literature directly describing the effect of USP5 on immune cells. Nakano et al. found that the inhibition of the USP5-induced Dll4 protein significantly reduced macrophage activation induced by uremic toxin indole sulfate [53]. USP5 was upregulated in macrophages infected with Salmonella typhimurium [2,54]. In addition, other members of the USP family have been found to have key roles in signaling in immune cells. For example, a study by Omilusik et al. found that USP1, an enzyme that belongs to the same USP family as USP5, is upregulated after T-cell activation and interacts with Id2 and Id3, which is essential for maintaining Id2 protein levels and the proliferation of memory CD8+ T-cells [55]. Although this study focused on USP1, it highlights the possible important role of USP family members in regulating T-cell function and memory cell responses. USP8 is essential for the development and maintenance of T-cells and affects the function of the T-cell antigen receptor (TCR) signalosome by interacting with Gad’s aptamer proteins and 14-3-3β regulatory molecules [56]. USP4 has been found to positively regulate IRF8 function in regulatory T-cells (Tregs) through K48-linked deubiquitination, thereby promoting the suppressive function of Tregs [56]. Although these studies did not directly focus on the role of USP5 in immune cell regulation, they highlighted the possible important role of USP family proteins in immune cell activation, differentiation, and function, providing a theoretical basis and research direction for future research on the role of USP5 in immune cell regulation.

### 3.2. USP5 Regulates the Immune Response

In the process of the immune response, NF-κB is responsible for regulating many aspects of innate and adaptive immune functions. It controls the expression of proinflammatory genes, including cytokines and chemokines, and plays a crucial role in the survival, activation, and differentiation of immune cells, especially innate immune cells and inflammatory T-cells [57,58]. In addition, NF-κB is essential in monocytes and macrophages, where it is involved in inflammasome activation, cytokine release, and cell survival by coordinating transcriptional and epigenomic programs to balance their phenotypes [59]. In this context, the deubiquitinating enzyme USP5, by affecting the stability and activity of key components in the NF-κB pathway, participates in the production of cytokines and the activation of immune cells and has an important impact on inflammation and the immune response [57,59]. Huang et al. found that USP5 promoted the expression of inflammatory cytokines by maintaining NF-κB signaling activation in human coronary artery endothelial cells [60]. Luo et al. found that USP5 stabilized TRAF6 by interacting with TRAF6 and removing its K48-linked polyubiquitin chain [61] (Figure 2F). This role is critical for the activation of NF-κB signaling and the subsequent production of proinflammatory cytokines. This suggests a role for USP5 in the regulation of NF-κB signaling and subsequent cytokine production, with potential implications for the development of inflammatory diseases such as rheumatoid arthritis [61]. Together, these studies reveal that USP5 plays an important role in regulating the immune response and inflammatory response by regulating the stability and activity of key proteins, thereby affecting the NF-κB signaling pathway and cytokine production.

In addition, USP5 plays an important role in the regulation of inflammatory cytokines and chemical factors in the process of the immune response, mainly through its effect on the production and function of tumor necrosis factor-α (TNF-α) [61]. TNF-α, a central inflammatory mediator, was decreased in USP5-knockdown rat basophilic leukemia (RBL-2H3) cells [2,54]. Qian et al. showed that USP5 plays a key role in TNF-α production through its specific deubiquitination, especially Smad ubiquitination regulatory factor 1 (Smurf1), which interacts with USP5. Moreover, it can promote the degradation of USP5 through the ubiquitin protease pathway and reduce the protein expression of USP5, thereby inhibiting the production of TNF-α [62] (Figure 2B). Palanisamy et al. found that USP5 may indirectly regulate cytokine mRNA stability and translation by affecting the function of RNA-binding proteins and microRNAs, thereby affecting the expression of cytokines such as TNFα, IL-6, and IL-8 [63]. Chen et al. showed that USP5 expression was positively correlated with the expression of the proinflammatory factors IL-1β, IL-6, and TNF-α [64]. Furthermore, other DUB family members, such as USP7, are known to regulate cytokine signaling by affecting the stability of immune signaling molecules, suggesting that USP5 may also be involved in the regulation of cytokine signaling through a similar mechanism [65]. In summary, although studies directly investigating how USP5 regulates specific cytokines are limited, the above studies suggest that USP5 may indirectly affect the production and function of inflammatory factors through a variety of mechanisms.

## 4. USP5 Regulates Immune Signaling Pathways to Affect the Development of Disease

### 4.1. Role of USP5 in Autoimmune Diseases

Autoimmune diseases, such as rheumatoid arthritis (RA), Kawasaki disease (KD), and systemic lupus erythematosus (SLE), are caused by abnormal immune system attacks on the normal tissues and cells of the body. Recent studies have shown that ubiquitin-specific protease 5 (USP5) plays an important regulatory role in the pathogenesis of these autoimmune diseases [60,61,66]. 

Luo et al. found that USP5 expression was significantly upregulated in the fibroblast-like synoviocytes (FLS) of RA patients compared to those of osteoarthritis (OA) patients. After IL-1β stimulation, the expression of USP5 increased in a time-dependent manner. The overexpression of USP5 significantly aggravated the production of proinflammatory cytokines and the associated activation of nuclear factor-κb (NF-κB) signaling, while the silencing of USP5 reduced the release of cytokines and inhibited the activation of NF-κB. In addition, USP5 interacts with tumor necrosis factor receptor-associated factor 6 (TRAF6) and removes its K48-linked polyubiquitinated chain, thereby stabilizing TRAF6. These data suggest that the positive regulation of USP5 in RA-FLS may be achieved by modulating the inflammatory process, suggesting that USP5 may be a potential target for RA treatment [61]. Similarly, in endothelial inflammation in Kawasaki disease (KD), USP5 acts as a positive regulator of TNFα-mediated immune responses in human coronary artery endothelial cells (HCAECs), and targeting USP5 to antagonate TNFα-mediated signaling provides a new target and therapeutic strategy for KD [60,67].

In addition, the IFN signaling pathway regulated by USP5 in SLE also has a key role [66,68]. Studies have shown that USP5 regulates the activation of IFN signaling through different mechanisms, thereby affecting the pathophysiological process of SLE. For example, Jian Yao et al showed that USP5 activates higher levels of IFN by increasing RIG-I protein levels [69]. Qian et al. found that USP5 physically interacts with Smurf1 and enhances the stability and turnover of Smurf1 by reducing its polyubiquitination level, thus, inhibiting IFN-activated p-STAT1 (the phosphorylation of the signal transducer and activator of transcription 1) [70]. In addition, the critical role IRF5 plays in inflammatory and autoimmune diseases may involve USP5-related regulatory mechanisms [71]. Lyn kinase was found to physically interact with IRF5 and inhibit the transcriptional activity of IRF5 by inhibiting its ubiquitination and phosphorylation. This mechanism helps to maintain immune homeostasis, and its abnormality may lead to the development of SLE-like diseases [72]. In addition, USP7 also plays an important role in SLE by stabilizing the expression of the IFNα receptor (IFNAR1) through deubiquitination, thereby affecting the IFNα signaling pathway and participating in the regulation of SLE disease activity [73]. Although the direct link between USP5 and SLE needs to be further studied, the regulatory role of USP5 in the IFN pathway and its interaction with other key factors provide a theoretical basis for exploring the mechanism of USP5 in SLE in the future. In summary, USP5, as an important regulatory protein, plays an important role in the pathogenesis of autoimmune diseases, which provides a new perspective for the treatment and pathological mechanism research of related diseases.

### 4.2. Role of USP5 in Inflammatory Immunity 

USP5 plays an important role in inflammatory immune regulation, especially its regulation of inflammation-related immune signaling pathways. Studies have found that USP5 was found to be upregulated in the gingival crevicular fluid and gingival tissues of patients with chronic periodontitis, and the expression level of USP5 was positively correlated with proinflammatory cytokines (such as TNF-α, IL-6, and IL-1β) [64,74]. This phenomenon suggests a potential function of USP5 in regulating inflammatory processes. In addition, the downregulation of USP5 and its related deubiquitinating enzyme activity inhibited the lipopolysaccharide (LPS)-induced inflammatory response of human periodontal ligament stem cells and also prevented the activation of the STAT3 signaling pathway [64]. These findings further suggest that USP5 is involved in the regulation of inflammatory responses by regulating specific signaling pathways. In other related studies, the role of USP5 has been further elucidated. For example, USP5 interacts with IKKβ to prevent IKKβ ubiquitination, which in turn inhibits the NF-κB signaling pathway, a mechanism proven to be effective in the MSCS treatment of acute lung injury [75,76] (Figure 2F). In addition, Cai et al. demonstrated that USP5 selectively promotes K48-linked NLRP3 polyubiquitination by recruiting the E3 ligase MARCHF7 and mediates its degradation through the autophagy lysosomal pathway [44] (Figure 2D). This finding provides new insights into the regulation of NLRP3 inflammasome hyperactivation and inflammatory innate immune responses by USP5. It is important to note that although much of the research has focused on USP5, Duan et al. provided insights into the role of a similar protein, USP3, in inflammatory immunity [77]. USP3 participates in the establishment of innate immune “tolerance” memory through a non-transcriptional feedback mechanism and negatively regulates TLR/IL1β-induced inflammatory signaling activation by specifically removing the K63-linked polyubiquitin chain on MyD88. At the same time, LPS pretreatment can induce the cytoplasmic retention of USP3 and a rapid and intense transfer to the cytoplasm upon a second LPS challenge, which allows USP3 to rapidly shut down NF-κB signaling [77]. These findings suggest that USP5 may be involved in inflammatory immune regulation through a similar mechanism and provide a new theoretical basis and future research direction. Taken together, USP5 plays a key role in inflammatory immunity and affects the inflammatory process by regulating immune signaling pathways. These studies provide important insights into understanding the role of USP5 in immune regulation and provide potential targets for the development of therapeutic strategies against inflammatory diseases.

### 4.3. Role of USP5 in Antiviral Immunity

More and more studies have found that USP5 also plays an important role in regulating antiviral immunity, especially by regulating IFN and interacting with other proteins, thereby initiating or weakening the antiviral immune response [78,79]. IFN is necessary for the host to combat viral infection by inducing innate immune responses, but the immune response must be tightly regulated to avoid self-harm [80]. For example, a study by Yao et al. in zebrafish further demonstrated that USP5 activates higher levels of IFN by increasing RIG-I protein levels, thereby achieving antiviral functions [69]. In addition, Jia et al. found that USP5 is a negative regulator of the IFN response induced by erythema viral neuro-necrosis virus (RGNNV) in sea bass [68]. The physical interaction of USP5 with Smurf1 was also found, and by reducing its polyubiquitination level, the stability and turnover of SMURF1 were enhanced, thereby inhibiting the expression of IFN-activated antiviral genes [70,81] (Figure 2B). These findings reveal a role for USP5 and the USP5-SmurF1 axis in regulating IFN-mediated antiviral activity. In addition, USP5 has been identified as an important regulator of IFN signaling in human embryonic kidney-293 T-cells [8]. Liu et al. conducted a systematic functional screening study and found that USP family proteins have broad and diverse roles in regulating antiviral responses. USP5 and other USP proteins were identified as factors that inhibit antiviral immunity at different levels, changing the ubiquitination status of the substrate by forming diverse signaling bodies with E3 ubiquitin ligases or USP rather than directly cutting the ubiquitin chain on the substrate through their protease activity [8]. In addition, Lin et al. found that USP25, a deubiquitinating enzyme similar to USP5, promotes IRF3 and NF-κB activation and subsequent type I interferon and proinflammatory cytokine production after viral infection by stabilizing TRAF3 and TRAF6 [82]. Wang et al. showed that USP4 positively regulates RIG-I-mediated antiviral responses via deubiquitylation and affecting RIG-I stability, thereby significantly enhancing RIG-I-triggered interferon beta (IFN-β) signaling and inhibiting viral replication [19]. Although these studies did not focus on USP25, the findings suggest a general mechanism by which deubiquitinating enzymes regulate antiviral immune responses, suggesting that USP5 may play a role in a similar regulatory process. These studies provide insight into the role of USP5 in regulating the IFN pathway and antiviral immune response, revealing the complex network through which it participates in regulating antiviral immunity through multiple mechanisms.

### 4.4. Role of USP5 in Tumor Immune Responses

The tumor immune response is a complex process in which USP5 has been found to play an important role in regulating key signaling pathways and protein stability in tumor cells. More and more studies have shown that USP5 affects tumor growth, metastasis, and escape from the immune response by targeting its substrates, such as p53, FoxM1, and β-catenin [2,49,83,84] (Figure 2).

In a variety of cancers, including pancreatic cancer, non-small cell lung cancer (NSCLC), and hepatocellular carcinoma (HCC), the upregulation of USP5 expression is associated with tumor development and deterioration [49,84,85]. For example, in pancreatic cancer, USP5 promotes tumor formation and progression by stabilizing the FoxM1 protein. Meanwhile, the overexpression of USP5 is associated with worsening tumor features, including large primary tumor size, poor differentiation, and high TNM stage, leading to significantly shorter overall survival times of patients [84]. Similarly, in NSCLC, USP5 acts as an oncogene to stabilize β-catenin through deubiquitination, activating the Wnt/β-catenin signaling pathway and promoting tumorigenesis [49,86] (Figure 2E). Pan et al. found that USP5 directly interacts with PD-L1 and deubiquitinates PD-L1 in NSCLC, thereby enhancing the stability of the PD-L1 protein (Figure 2E). This contributes to the immune escape of cancer cells, thereby promoting the development and progression of NSCLC [87]. In addition, Cao et al. also found that USP5 knockdown alleviated lung cancer progression by activating the PARP1-mediated mTOR signaling pathway in a 2023 study [88]. In HCC, the upregulation of USP5 is associated with tumor growth, drug resistance, and reduced apoptosis, suggesting its role in carcinogenesis and as a potential target for HCC therapy [85]. In addition, USP5 also plays an important role in promoting breast cancer cell proliferation and metastasis. Studies have shown that USP5 affects signal transduction and disease progression by stabilizing the hypoxia-inducible factor 2α (HIF2α) protein and promoting the transcription of HIF2α target genes, such as SLC2A1, PLOD2, P4HA1, and VEGFA [89]. Notably, the interaction of USP7 with mixed lineage leukemia 5 protein (MLL5) was also found to enhance MLL5 stability, thereby affecting the expression level of MLL5 in the nucleus [47] (Figure 2E).

These findings reveal the important role of USP5 in regulating key signaling pathways and protein stability in tumor cells, which in turn affect tumor growth, metastasis, and immune escape.

## 5. Therapeutic Developments for Targeting USP5

In view of the important role of USP5 in maintaining protein stability, regulating related immune signal transduction, and the above-mentioned various tumoral, inflammatory, and antiviral processes, the targeted therapy of USP5 has become a new research hotspot [2,10,90]. For example, Li et al. showed that USP5 inhibition could significantly slow the growth of pancreatic tumors and promote the tumorigenesis and progression of pancreatic cancer by stabilizing the FoxM1 protein, which provided a basis for USP5 as a potential target for PDAC treatment [84]. Alternatively, Mann et al. developed a series of USP5 inhibitors that demonstrated the selective inhibition of USP5 by competitively inhibiting the catalytic activity of USP5 and binding to the ZnF-UBD of USP5 [40]. This study provides a chemical and structural framework and a chemical probe for the further exploration of USP5 function. These studies provide potential research directions and application potential for the development of USP5 inhibitors and further exploration of USP5 in the treatment of cancer and other diseases. In addition, several USP5 inhibitors (Table 1) are being developed for the treatment of related diseases. For example, WP1130 is a current and commonly used USP5 inhibitor in USP5-related studies, and it has also been investigated as a cancer therapeutic agent [10,91]. WP1130 is a partially selective DUB inhibitor that inhibits the activity of a variety of DUBs, including USP5, USP9X, USP14, and USP37, and increases K63 and K48-mediated polyubiquitin chains [91,92]. Kapuria et al. showed that WP1130 inhibited USP5 activity by inducing the upregulation of the proapoptotic protein p53 [92]. WP1130 also reduced tumorigenicity and metastasis in a xenograft mouse model by inhibiting USP5 activity and degrading the WT1 protein [93]. In addition, EOAI is a pan-deubiquitinase inhibitor that targets USP5. Zheng et al. found that EOAI could effectively induce DNA damage, cell cycle arrest, apoptosis, and autophagy in NSCLC cells both in vivo and in vitro [94]. It also enhances the antitumor effect of cisplatin, suggesting its potential as a combination therapy for NSCLC [95]. These studies show that USP5 inhibitors have important effects on tumor growth and development by directly inhibiting the growth of tumor cells by regulating the stability of tumor suppressor proteins and enhancing antitumor immune responses through immunoregulatory mechanisms, suggesting the potential of strategies targeting USP5 in cancer treatment.

## 6. Discussion and Prospects

In summary, this article reviews the structure and function of USP5 in immune regulation, the regulation of immune signal transduction, and the potential of targeted therapy. Taken together, USP5 plays a key role in the regulation of the immune response and immune signaling pathways that affect the occurrence and development of diseases. It affects the activity of immune cells and immune responses by regulating protein stability and many signal transduction pathways, such as Wnt/β-catenin, NF-κB, and IFN, thus regulating the occurrence and development of immune-related diseases. In addition, USP5 also plays an important role in tumor growth, treatment resistance, and immune escape and has potential anticancer therapeutic value. In summary, a deep understanding of the mechanism of USP5 is of great significance for revealing its immune regulation and disease pathogenesis mechanisms and developing new therapeutic strategies.

Although the current research on USP5 in immune regulation and disease pathogenesis has made some progress, indicating its key role in regulating protein stability and signal transduction pathways, the specific mechanism of action of USP5 still needs to be further explored, especially the mechanism and regulatory network in different disease states. In addition, the research on USP5 as a therapeutic target is still in its preliminary stage and its feasibility and effectiveness in treatment need to be further verified. In addition, due to the complexity of immune regulation and disease pathogenesis, it is necessary to overcome the limitations of experimental models and the lack of technical means in order to more comprehensively reveal the mechanism of USP5 in this field. Therefore, despite some progress, more studies are needed to address the current dilemma to fully understand the role of USP5 in immune regulation and disease pathogenesis.

## Figures and Tables

**Table 1:** USP5 inhibitors as potential target drugs.

Inhibitor	Target	Mechanism	Diseases	References
WP1130	USP5/9X/14/24/37	Inhibition of USP5 activity by inducing the upregulation of the proapoptotic protein p53	Cancers	[29,91,92]
PYR-41	USP5/9X	PYR-41 reduced USP5 protein levels in a dose-dependent manner by inducing protein cross-linking to form high molecular weight complexes	Lymphoma	[96]
Formononetin	USP5	Formononetin directly acts on USP5 to destabilize SLUG and inhibit EMT in HCC	HCC	[97,98]
		The inhibition of USP5 increases the degradation of FcεRI signaling and induces FcεRIγ ubiquitination, thereby inhibiting IgE-mediated mast cell activation	Allergic inflammation	[98]
II-1, Suramin, Gossypetin	USP5	The inhibition of the biochemical interaction between USP5 and the Cav3.2 domain III-IV linker ultimately reduces pain-related information transmission	Inflammatory and neuropathic pain	[99,100]
Mebendazole	USP5	The inhibition of USP5 expression and disruption of the interaction between USP5 and c-Maf results in increased c-Maf ubiquitination and subsequent c-Maf degradation	Multiple myeloma	[42,101]
EOAI	USP5/9X/14/24/	EOAI is a pan-deubiquitinase inhibitor that targets USP5	Pancreatic ductal adenocarcinoma, NSCLC	[10,29,94,102]
Vialinin A	USP4/5	Vialinin A inhibits the enzymatic activity of USP5 and inhibits the ability of USP5 to hydrolyze Ub-AMC in a dose-dependent manner	Inflammation	[67]
